# The impact of demographic, anthropometric and athletic characteristics on left atrial size in athletes

**DOI:** 10.1002/clc.23368

**Published:** 2020-04-09

**Authors:** Georgios A. Christou, Jamie M. O'Driscoll

**Affiliations:** ^1^ Laboratory of Sports Medicine, Sports Medicine Division Aristotle University of Thessaloniki Thessaloniki Greece; ^2^ MSc Sports Cardiology St George's University of London London UK; ^3^ School of Human and Life Sciences Canterbury Christ Church University Canterbury UK; ^4^ Department of Cardiology St George's Healthcare NHS Trust London UK

**Keywords:** age, body surface area, endurance sports, ethnicity, gender, lean body mass, left atrial diameter, left atrial volume, left atrium, training age

## Abstract

The structural adaptations of the “athlete's heart” include left atrial (LA) enlargement. A literature search was performed based on PubMed listings up to November 2, 2019 using “athletes AND left atrium,” “athletes AND LA,” “sports AND left atrium,” “sports AND LA,” “exercise AND left atrium,” and “exercise AND LA” as the search terms. Eligible studies included those reporting the influence of demographic, anthropometric and athletic characteristics on LA size in athletes. A total of 58 studies were included in this review article. Although LA volume has been reported to be greater in males compared to females when indexed for body surface area (BSA), there was no difference between sexes. The positive association between LA size and age in athletes may reflect the increase in body size with maturation in nonadult athletes and the training age of endurance athletic activity in adult athletes. Caucasian and black athletes have been demonstrated to exhibit similar LA enlargement. The positive association of LA size with lean body mass (LBM) possibly accounts for the relationship of LA size with BSA. LA enlargement has been reported only in endurance‐trained, but not in strength‐trained athletes. LA size appears to increase with an increase in both the volume and intensity of endurance training. LA size correlates independently with the training age of endurance athletes. The athlete's characteristics that independently determine LA size include LBM, endurance training, and training age.

## 1 INTRODUCTION

It has been well established that chronic exercise training results in specific cardiac adaptations, collectively known as the “athlete's heart.”^1^ These cardiac adaptations include electrical, structural and functional alterations that are generally considered benign.^2^, ^3^, ^4^ With regard to structural adaptations, there is evidence of cardiac chamber enlargement, increased left ventricular (LV) wall thickness, and prominent LV trabeculations.^3^, ^4^ Notably, increased cavity size appears to involve both the left and right heart and it has been shown to affect not only the ventricles, but the atria as well.^3^, ^4^

Growing concerns have arisen regarding the left atrial (LA) enlargement encountered in athletes, in the light of the potential adverse consequences attributed to increased LA size, including predisposition to atrial arrhythmias and especially atrial fibrillation.^5^, ^6^ Although LA enlargement in athletes can be reasonably considered benign in the absence of increased LV filling pressures and mitral valve dysfunction, the excessive LA enlargement in some athletes may challenge the notion of the benign nature of this process. In this respect, the characteristics of the athlete predisposing to LA enlargement are important to be elucidated, since this information will enable the clinician to judge whether the magnitude of LA enlargement in an athlete can be normally expected on the basis of the athlete's characteristics. Furthermore, the importance of the study of characteristics of the athletes with LA enlargement relies on the identification of possible modifiable predisposing characteristics leading to LA enlargement, such as training volume, that, if changed, could decrease the severity of LA enlargement in the case of athletes with atrial arrhythmias. Therefore, this review article discusses the demographic, anthropometric, and athletic characteristics associated with LA enlargement and attempts to elucidate the relative importance of them in determining LA size in athletes. No previous study has addressed these issues.

## 2 METHODS

### 2.1 Search strategy

**FIGURE 1:**
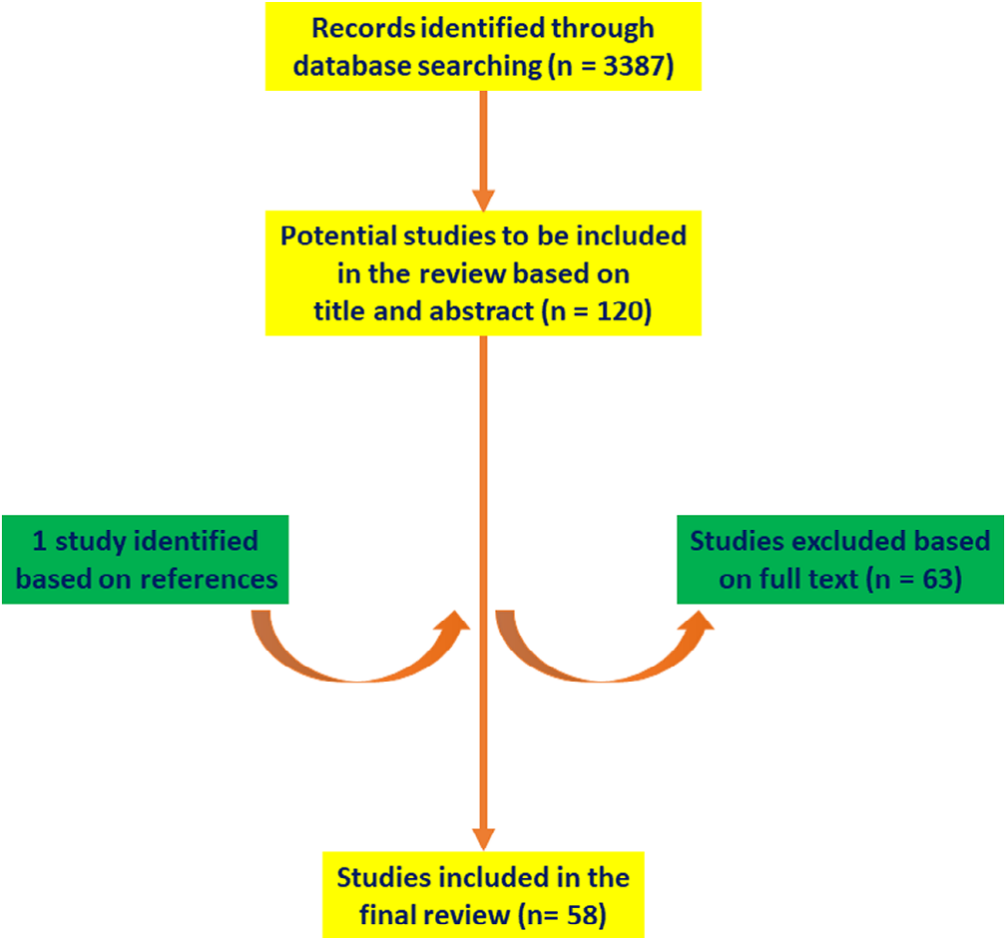
Schematic flowchart for the selection of studies to be included in the review

A literature search based on PubMed listings up to November 2, 2019 using “athletes AND left atrium,” “athletes AND LA,” “sports AND left atrium,” “sports AND LA,” “exercise AND left atrium,” and “exercise AND LA” as the search terms identified 3387 articles (Figure 1). The reference lists of these articles were also interrogated and articles that were judged relevant were selected for inclusion in the review. In total, 58 studies were included in the text of this review article.

### 2.2 Inclusion criteria

Studies fulfilling the following criteria were considered eligible for inclusion in the review:

1. Original research articles.
2. Studies investigating human individuals.
3. Studies mentioning the influence of demographic, anthropometric or athletic characteristics on LA size in athletes.
4. Full text written in English.

## 3 THE INFLUENCE OF ATHLETE'S CHARACTERISTICS ON LA SIZE

Table 1 summarizes the characteristics and key findings of the main studies included in this review article. The main studies that were deemed eligible to be included in Table 1 were the ones fulfilling the following criteria: (a) at least one study investigating the effect of each individual athlete's characteristic on LA size, (b) studies with results that best reflect the effects of athlete's characteristics on LA size, (c) studies with novel results not investigated by other studies, and (d) among studies reporting similar information, we selected to include the study with the greatest number of participant.

**TABLE 1:** The main studies evaluating the effects of demographic, anthropometric and athletic characteristics on left atrial size in athletes

Studies	Athletic study population	Findings
Prakken et al^8^	120 endurance athletes 60 regular athletes: training volume 9‐18 h/wk 60 elite athletes: training volume > 18 h/wk	Elite athletes vs regular athletes: ↑ LAV Multiple regression analysis. Independent predictors of LAV: BSA, training volume, gender
Giraldeau et al^9^	90 Caucasian college athletes (45 males, 45 females)	Males vs females: ↑ LAV, ↔ LAV/BSA, ↔ LAV/LBM^0.7^
Wilhelm et al^10^	138 athletes (70 males, 68 females)	Males vs females: ↑ LAV, ↔ LAV/BSA
George et al^11^	464 junior athletes (14‐18 y old)	Males vs females: ↑ LAD Athletes 17‐18 y old vs athletes 14 y old: ↑ LAD Correlation with LAD: body weight (*b* exponent = 0.3 ± 0.1), BSA (*b* exponent = 0.5 ± 0.2)
Lakatos et al^13^	138 athletes (85 males, 53 females)	Males vs females: ↔ LAV/BSA (3D echocardiography)
Nistri et al^15^	157 athletes	Correlation with LAV/BSA: male gender, BMI (*r* > 0), age (*r* > 0)
D'Andrea et al^16^	615 athletes 370 endurance‐trained 245 strength‐trained	Endurance‐trained athletes vs strength‐trained athletes: ↑ LAD, ↑ LAV/BSA Correlation with LAV/BSA: age (*r* > 0), male gender, training age (*r* > o), endurance training Multiple regression analysis. Independent predictors of LAV/BSA: endurance training, training age
Legaz Arrese et al^17^	188 runners (134 males, 54 females) 57 sprint‐trained 73 middle distance‐trained 58 long distance‐trained	Long distance‐trained vs middle distance‐trained: ↔ LAD (males), ↔ LAD (females) Long distance‐trained vs sprint‐trained: ↑ LAD (males), ↔ LAD (females) Middle distance‐trained vs sprint‐trained: ↑ LAD (males), ↔ LAD (females)
Cavarretta et al^20^	2261 peri‐pubertal athletes	Correlation with LAD: age (*r* > 0), BSA (*r* > 0) Multiple regression analysis. Independent predictor of LAD: BSA
Somauroo et al^21^	172 teenage soccer players	Correlation with LAD: BSA (*r* > 0), not with age (*P* = .100)
Elliott et al^25^	96 endurance athletes. Three groups according to lifetime training hours L: (n = 30) < 3000 h M: (n = 31) 3000‐6000 h H: (n = 35) > 6000 h	H vs L: ↑ LAV/BSA, ↔ LAD M vs L: ↑ LAV/BSA, ↔ LAD
Gjerdalen et al^29^	553 football players (504 Caucasian, 49 African)	Caucasian vs African: ↔ LAV, ↔ LAV/BSA, ↔ LAD
Riding et al^30^	1166 athletes (596 Arabic, 410 Black African, 160 Caucasian)	Caucasian vs Arabic: ↑ age, ↑ BSA, ↑ LAV, ↑ LAD Caucasian vs Black: ↑ age, ↑ BSA, ↔ LAV, ↔ LAD Black vs Arabic: ↑ age, ↑ BSA, ↑ LAV, ↑ LAD
McClean et al^37^	36 male athletes 18 high dynamic‐high static 18 low dynamic‐high static 20 male age‐matched controls	High dynamic‐high static vs Low dynamic‐high static: ↑ LAV, ↑ LAD High dynamic‐high static vs controls: ↑ LAV, ↑ LAD Low dynamic‐high static vs controls: ↔ LAV, ↔ LAD
Kooreman et al^42^	72 female college athletes (n = 37) higher intensity group (n = 35) lower intensity group 31 female, age‐matched controls	Higher intensity group vs controls: ↑ LAV/BSA Lower intensity group vs controls: ↔ LAV/BSA Higher intensity group vs Lower intensity group: ↑ LAV/BSA, ↑ LAV/LBM
D'Andrea et al^45^	650 athletes (n = 395) endurance‐trained (n = 255) strength‐trained 230 age‐, gender‐matched controls	Endurance‐trained vs controls: ↑ LAV/BSA Strength‐trained vs controls: ↔ LAV/BSA Endurance‐trained vs strength‐trained: ↑ LAV/BSA
Dores et al^54^	108 athletes 60 high level (training volume ≥ 20 h/wk) 48 low level (training volume < 10 h/wk)	High level athletes vs low level athletes: ↑ LAV/BSA
Mahjoub et al^57^	17 male endurance‐trained athletes	High intensity interval training with cycle ergometer (3 sessions/wk) for 6 wk → ↑ LAV
Król et al^58^	114 rowers (61 males, 53 females)	Correlation with LAV: VO_2max_ (*r* > 0)
D'Ascenzi et al^62^	21 adolescent soccer players after 2 mo of detraining	After 4 mo of exercise training vs baseline: ↑ LAV/BSA After 8 mo of exercise training vs baseline: ↑ LAV/BSA
Pedlar et al^66^	21 runners having participated in Boston Marathon	After 8 wk of detraining vs peak prerace performance: ↔ LAV
Hasdemir et al^72^	23 retired football players (>50 y old) 18 controls (>50 y old). Never regular exercise in their life	Retired athletes vs controls: ↑ LAD

*Note:* Symbols “↑” and “↔” represent increase and no difference, respectively.

Abbreviations: BSA, body surface area; LAD, left atrial diameter; LAV, left atrial volume; LBM, lean body mass; VO_2max_, maximal oxygen uptake.

### 3.1 Gender

The majority of studies have shown that male athletes have greater LA volume compared to female athletes.^7^, ^8^, ^9^, ^10^ Moreover, George et al demonstrated that among nonadult athletes, LA diameter was increased in males compared to females.^11^ Taking into account that LA enlargement usually occurs asymmetrically and LA diameter has been demonstrated to underestimate LA volume especially in females, the comparison of LA size between male and female athletes is more appropriately performed using LA volume rather than LA diameter.^12^ However, the difference in LA volume between male and female athletes has been reported to disappear after indexing for body surface area (BSA) or lean body mass (LBM).^9^, ^10^, ^13^, ^14^ Considering that females are normally characterized by greater body fat percentage, the persistence in few studies of the difference in LA volume between male and female athletes after indexing for BSA may not represent a valid analysis.^7^, ^15^, ^16^ In this case, indexing for the metabolically active LBM is possibly more appropriate. Therefore, the greater LBM of male athletes may account at least in part for their greater LA size.

Nevertheless, the presence of residual confounding cannot be totally excluded in the comparison of LA volume indexed for BSA between male and female athletes, since the training regimens of male athletes are usually characterized by greater volume and intensity, which may in turn affect LA size to a greater extent. Indeed, Mosen et al demonstrated that LA volume indexed for BSA differed only between male and female athletes, but not between male and female controls.^7^ Moreover, Legaz Arrese et al reported that LA diameter of long‐ and middle‐distance runners was increased compared to sprint runners; however, this was only true in male athletes.^17^ The comparison of LA size between male and female athletes needs further investigation, and importantly, comparisons need to be made between genders matched for sporting discipline and training volume and intensity, and indexing LA volume to LBM, producing body size independent measures.

### 3.2 Age

With regard to the relationship between LA size and age in athletes, LA enlargement has been shown to be more pronounced in adult athletes compared to teenage athletes of the same sport.^18^, ^19^ In addition, LA size in athletes has been found to increase with increasing age from childhood to early adulthood.^11^, ^20^ However, the relationship between LA size and age from childhood to early adulthood appears to result from the accompanied increase in body size with maturation, rather than from an independent effect of chronological age per se, since the relationship between LA size and age has been found to disappear after adjustment for body size.^19^, ^20^ Even more, Somauroo et al showed that among teenage soccer players LA diameter correlated only with BSA and not with age.^21^

In the majority of studies, a positive association between LA size and age has been found in adult athletes.^13^, ^15^, ^22^, ^23^, ^24^ Taking into account that this relationship has been shown to be attenuated after adjustment for training age, this association in athletes possibly occurs at least in part through the effect of training age on LA size.^16^, ^25^ Indeed, the LA has been reported to progressively enlarge over the years of active athletic life in endurance‐trained athletes.^24^, ^26^ In other words, the greater number of lifetime endurance training hours of older athletes may determine their greater LA size. Consistently, LA size in healthy nonathletes has been reported to be determined mostly by body size rather than age and thus an increase in LA size is possibly an expression of pathology and it cannot be attributed to normal aging.^27^, ^28^

### 3.3 Ethnicity

The overwhelming majority of studies evaluating LA size in athletes is in Caucasian athletic populations. Few studies have investigated whether LA size in athletes is influenced by ethnicity.^29^, ^30^, ^31^ Specifically, Caucasian and black athletes have been demonstrated to exhibit similar LA enlargement, while Caucasian athletes were characterized by greater LV size and decreased relative wall thickness compared to black athletes.^29^, ^30^, ^31^ Thus, black athletes may have disproportionately greater LA size compared to LV enlargement. A possible explanation for these findings may be the equally thin atrial wall of both Caucasian and black athletes permitting similar LA enlargement, as opposed to the greater LV wall thickness of black athletes limiting the magnitude of LV dilatation compared to Caucasian athletes.

With regard to Arabic athletes, Riding et al showed that these athletes had decreased LA size compared to both Caucasian and black athletes.^30^ However, in this study, Arabic athletes were younger and had a lower BSA compared to Caucasian and black athletes and the authors used measures of LA size without adjustment for body size.^30^ Thus, the reported differences in LA size may have been confounded by the different ages and BSA between the athletes.^30^ Further studies are needed to elucidate whether LA size differs between athletes of different ethnicity, who are matched for gender, age, body size, and sport discipline.

### 3.4 Anthropometric characteristics

LA size, expressed as either LA anteroposterior diameter or LA volume, has been demonstrated to correlate positively with BSA.^7^, ^8^, ^20^, ^22^, ^32^ Taking into account that BSA represents an estimate of the total tissue mass perfused and hence of cardiac output, this association of LA size with BSA possibly reflects the impact of cardiac volume overload on LA remodeling.^33^ Consistently, both LA and LV enlargement in athletes have been found to be balanced with a strong independent correlation between them, mirroring the same magnitude of loading conditions.^15^ According to the law of Laplace (ie, wall tension = intracavitary pressure × cavity radius/[2 × wall thickness]), the thinner LA wall is associated with higher wall tension predisposing the LA to dilatation, while the LA enlargement in athletes without concomitant increases in LA wall thickness further increases LA wall tension promoting additional LA enlargement.

Scaling of LA size should be ideally performed for LBM, which is the metabolically active tissue mass in human body.^34^ Scaling for body weight, as a proxy measure of LBM, can be precise to the extent that the body fat percentage remains similar across the study population, which is not always the case among athletes of different sports.

Importantly, a common pitfall in allometric scaling of cardiovascular parameters is the inappropriate power to which is raised the allometric parameter used for the scaling of cardiovascular parameters.^34^ The ideal scaling procedure should result in body size independent variables.^34^ Even more, based on the theory of geometric similarity the one‐dimensional LA diameter should be appropriately indexed for LBM^0.3^ and BSA^0.5^, while the three‐dimensional LA volume for LBM and BSA^1.5^.^11^, ^34^, ^35^ Consistently, LA volume indexed for BSA may not be body size independent, as indicated by the reported association between this variable and body mass index in both athletes and nonathletes.^15^ Appropriately powered prospective studies are needed to investigate this issue further, such as ascertaining any changes in LA size following weight loss interventions in athletes and nonathletes. With regard to nonadult athletes, the relationship between LA size and BSA appears to underly the increase in LA size with aging during maturation.^11^, ^20^, ^21^, ^36^

### 3.5 Sport discipline

The degree of LA enlargement in athletes has been found to depend on sport discipline. Specifically, only endurance‐trained athletes have been reported to have increased LA size compared to nonathletes, while the LA size of strength‐trained athletes has been shown to be similar to age‐ and gender‐matched controls.^16^, ^37^, ^38^, ^39^, ^40^, ^41^, ^42^, ^43^, ^44^, ^45^, ^46^ Of particular interest, there appears to be a more pronounced LA enlargement reported in endurance cyclists.^37^, ^38^, ^40^, ^47^ In addition, among athletes performing the same mode of exercise, LA size appears to increase as the endurance component of the sport increases.^17^, ^48^ Indeed, LA size was found to be greater in both long‐ and middle‐distance runners compared to sprint runners.^17^ Similarly, LA size of baseline tennis players was shown to be increased compared to offensive tennis players, who are known to be characterized by lower energy demands during training and competition compared to the former.^48^, ^49^

The effect of endurance training on LA size is possibly mediated through the volume overload of this type of training, resulting in a balanced enlargement of all cardiac cavities.^15^, ^16^, ^42^, ^50^, ^51^, ^52^ Indeed, the ratio of LA volume to LV end‐diastolic volume has been shown not to differ among endurance‐trained athletes, strength‐trained athletes and controls.^42^

With regard to sports in which a high body weight confers an advantage to the athlete, such as the position of lineman in American Football, athletes often seek advice for weight gain with the goal of becoming larger than their opponents. These athletes are characterized by greater LA size compared to other type of athletes, while this difference has been found to disappear after indexing for BSA, implying that this difference can be attributed to the greater body size of the former.^53^

### 3.6 Training regimen

Both training volume and intensity appear to determine the effect of endurance exercise training on LA enlargement. The impact of training volume on LA size has been more adequately studied compared to the effect of training intensity. Specifically, among endurance‐trained athletes, higher training volumes have been associated with greater LA size.^8^, ^54^, ^55^ This pattern was demonstrated to exist even in junior athletes.^56^ Regarding the effect of training intensity on LA size, Mahjoub et al demonstrated that LA volume in endurance athletes increased following 6 weeks of high intensity interval training with cycle ergometer.^57^

The reported positive association between LA size and maximal oxygen uptake in athletes can be possibly explained by the fact that higher aerobic capacity may reflect a higher level of endurance training, which in turn can contribute to LA enlargement.^7^, ^40^, ^58^, ^59^, ^60^, ^61^ With regard to the association between LA size and maximal oxygen uptake, when the latter is indexed for body weight, this relationship possibly reflects only the level of endurance training, but when the latter is expressed in absolute terms (ie, L/min) this association may incorporate information about both endurance training and body size.

Importantly, LA size has been shown to increase progressively during the process of a training macrocycle from preseason to end season.^62^, ^63^, ^64^, ^65^ However, LA volume in marathon runners has been found not to change following 2 months of detraining from peak prerace performance.^66^ Therefore, the evaluation of LA size in athletes should consider the current training status of the athletes with regard to not only the current training regimen, but also the exact phase in the temporal sequence of training periodization.

The increase in LA size during the process of a training macrocycle appears to be associated with a decline in peak reservoir and contraction LA strains after at least 4 months of intensification of exercise training.^32^, ^57^, ^62^ The clinical significance of this exercise training‐associated decline in LA strain remains to be elucidated.

### 3.7 Training age

Among endurance‐trained athletes, training age, as estimated by lifetime training hours, lifetime years of training or total number of previous competitions, has been demonstrated to correlate positively with LA size.^16^, ^25^, ^50^, ^67^ Consistently, the LA size of endurance athletes has been shown to progressively increase over the years of their athletic career.^26^ However, Brugger et al reported that peak reservoir, conduit and contraction LA strains (LA mechanics) did not differ among three groups of amateur male runners stratified according to low, moderate and high lifetime training hours.^67^

Considering that former endurance athletes have been reported to have greater LA size compared to age‐ and gender‐matched nonathletes, the adaptation of LA size to endurance training appears to be long lasting with residual LA enlargement many years after the end of the athletic career.^68^, ^69^, ^70^, ^71^, ^72^ Notably, this residual training effect on LA size appears to be at odds with the reversible changes in LV wall thickness and LV end‐diastolic volume following a long‐term deconditioning period.^70^, ^72^, ^73^

Thus, LA size in endurance athletes is possibly determined by the lifetime exposure to volume overload in the context of endurance exercise training with the additive effect of current endurance training to further enhance the exercise‐induced adaptations of LA size.

## 4 KNOWLEDGE GAPS AND FUTURE AVENUES FOR RESEARCH

The comparison of LA size between male and female athletes needs to be investigated between participants matched for sport discipline and training volume and intensity, as well as indexing LA volume to LBM. Further studies are needed to elucidate whether LA size differs between athletes of different ethnicity, who are matched for gender, age, body size and sport discipline. In addition, the impact of exercise intensity on LA size should be further investigated by altering training frequency and/or training intensity of the workouts of interval training.

The overwhelming majority of studies, which have evaluated the impact of athlete's characteristics on LA size used 2D echocardiography. Cardiac magnetic resonance (CMR) imaging represents a more accurate and reproducible method for the evaluation of cardiac volumes.^8^ Specifically, measurement of LA volume with 2D echocardiography is limited by foreshortened views and geometric modeling resulting in decreased LA volumes compared to CMR imaging.^74^, ^75^ Therefore, further studies are needed using CMR imaging in the evaluation of LA volumes, especially to confirm whether an athlete's characteristic so far considered to have a neutral effect on LA size, such as ethnicity, has indeed no effect.

Finally, the incorporation of myocardial mechanical analysis using LA strain can aid in the detection of abnormal LA function even in athletes with normal LA size.^76^, ^77^ Importantly, impaired LA strain has been demonstrated to predict not only the development of atrial fibrillation, but atrial fibrillation‐related stroke as well, over and above clinical and standard echocardiographic parameters.^78^, ^79^ The predictive value of LA strain for the development of atrial fibrillation has also been reported to exist in male endurance veteran athletes.^80^ Interestingly, in patients with a normal LA size, LA strain was more predictive of atrial fibrillation, whereas in patients with abnormal LA volumes, LV strain was more predictive of atrial fibrillation.^78^ Therefore, it is conceivable that both LA and LV strain (global atrioventricular strain) and associated interrogation of the LA‐LV coupling, which provides a comprehensive assessment of left heart mechanics, may contribute to the identification of benign or adverse remodeling that may predispose to the development of atrial fibrillation. In this respect, future studies investigating the impact of athlete's characteristics on LA size should use strain analysis of both LA and LV, in order to elucidate whether the effect of each characteristic on LA size in athletes has any prognostic relevance in terms of predisposition to atrial fibrillation.

## 5 CONCLUSIONS

**FIGURE 2:**
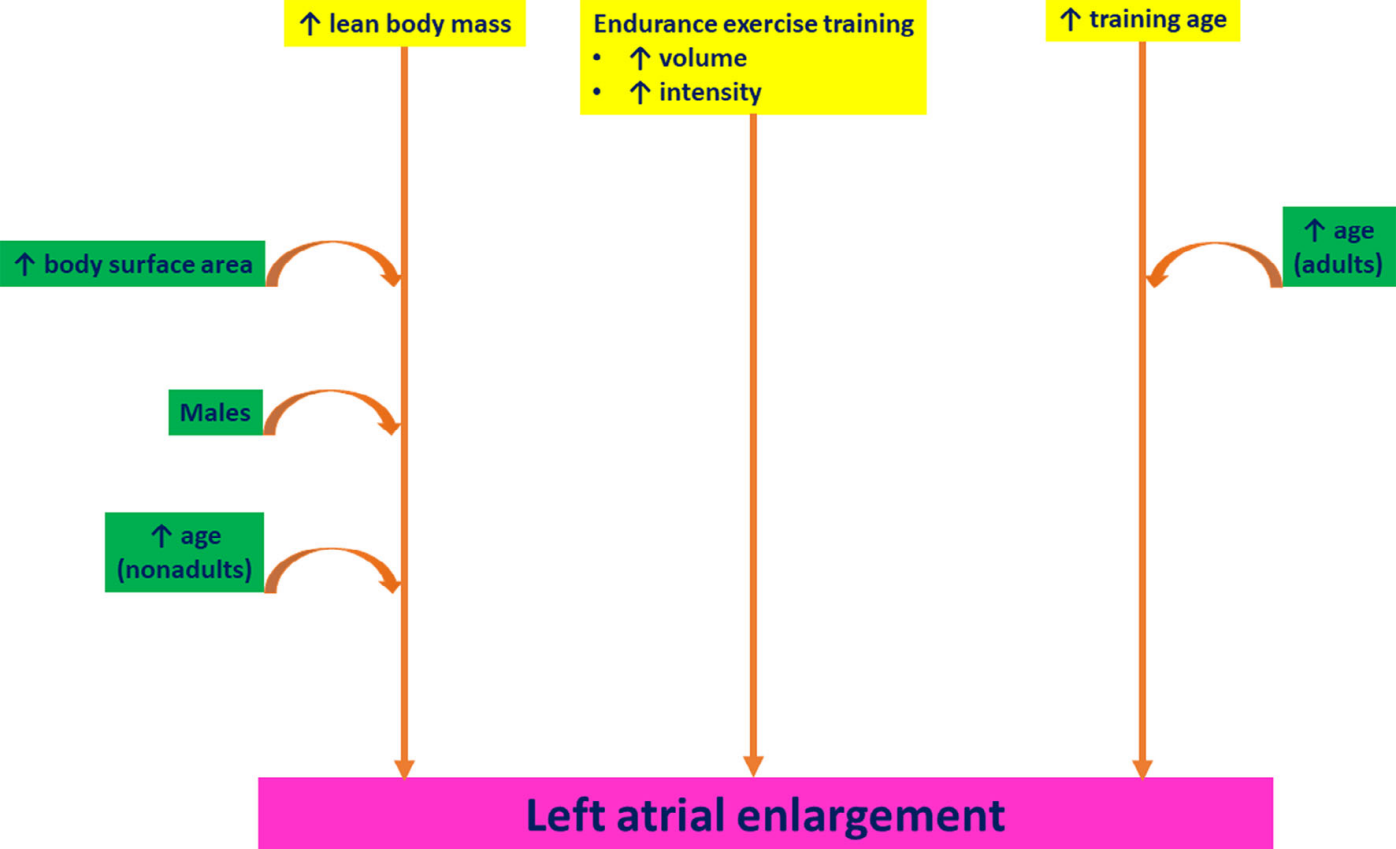
The direct and indirect effects of demographic, anthropometric and athletic characteristics on left atrial size in athletes. The straight arrows indicate direct effects, while the curved arrows suggest indirect associations

LA enlargement appears to be a well‐established component of the “athlete's heart.” The athlete's characteristics that independently determine LA size include LBM, endurance training and training age (Figure 2). The effects of gender, age, and ethnicity on LA size in athletes may be mediated through the above‐mentioned characteristics. The accurate interpretation of these variables, which impact LA size in athletes, is of significant clinical importance, in order to ascertain if the LA enlargement detected in an athlete is physiological or represents an underlying cardiac disease.

## CONFLICT OF INTEREST

The authors declare no potential conflict of interests.

## References

[1] Maron BJ , Pelliccia A . The heart of trained athletes: cardiac remodeling and the risks of sports, including sudden death. Circulation. 2006;114(15):1633‐1644. 17030703 10.1161/CIRCULATIONAHA.106.613562

[2] Sharma S , Drezner JA , Baggish A , et al. International recommendations for electrocardiographic interpretation in athletes. J Am Coll Cardiol. 2017;69(8):1057‐1075. 28231933 10.1016/j.jacc.2017.01.015

[3] Oxborough D , Augustine D , Gati S , et al. A guideline update for the practice of echocardiography in the cardiac screening of sports participants: a joint policy statement from the British Society of Echocardiography and Cardiac Risk in the Young. Echo Res Pract. 2018;5(1):G1‐G10. 29551755 10.1530/ERP-17-0075 PMC5861331

[4] Pelliccia A , Caselli S , Sharma S , et al. European Association of Preventive Cardiology (EAPC) and European Association of Cardiovascular Imaging (EACVI) joint position statement: recommendations for the indication and interpretation of cardiovascular imaging in the evaluation of the athlete's heart. Eur Heart J. 2018;39(21):1949‐1969. 29029207 10.1093/eurheartj/ehx532

[5] Andersen K , Farahmand B , Ahlbom A , et al. Risk of arrhythmias in 52 755 long‐distance cross‐country skiers: a cohort study. Eur Heart J. 2013;34(47):3624‐3631. 23756332 10.1093/eurheartj/eht188

[6] Abdulla J , Nielsen JR . Is the risk of atrial fibrillation higher in athletes than in the general population? A systematic review and meta‐analysis. Europace. 2009;11(9):1156‐1159. 19633305 10.1093/europace/eup197

[7] Mosén H , Steding‐Ehrenborg K . Atrial remodelling is less pronounced in female endurance‐trained athletes compared with that in male athletes. Scand Cardiovasc J. 2014;48(1):20‐26. 24279839 10.3109/14017431.2013.860234

[8] Prakken NH , Velthuis BK , Bosker AC , et al. Relationship of ventricular and atrial dilatation to valvular function in endurance athletes. Br J Sports Med. 2011;45(3):178‐184. 19687209 10.1136/bjsm.2009.059188

[9] Giraldeau G , Kobayashi Y , Finocchiaro G , et al. Gender differences in ventricular remodeling and function in college athletes, insights from lean body mass scaling and deformation imaging. Am J Cardiol. 2015;116(10):1610‐1616. 26456207 10.1016/j.amjcard.2015.08.026

[10] Wilhelm M , Roten L , Tanner H , Wilhelm I , Schmid JP , Saner H . Gender differences of atrial and ventricular remodeling and autonomic tone in nonelite athletes. Am J Cardiol. 2011;108(10):1489‐1495. 21864814 10.1016/j.amjcard.2011.06.073

[11] George K , Sharma S , Batterham A , et al. Allometric analysis of the association between cardiac dimensions and body size variables in 464 junior athletes. Clin Sci (Lond). 2001;100(1):47‐54. 11115417

[12] Nikitin NP , Witte KK , Thackray SD , et al. Effect of age and sex on left atrial morphology and function. Eur J Echocardiogr. 2003;4(1):36‐42. 12565061 10.1053/euje.2002.0611

[13] Lakatos BK , Molnár AÁ , Kiss O , et al. Relationship between cardiac remodeling and exercise capacity in elite athletes: incremental value of left atrial morphology and function assessed by three‐dimensional echocardiography. J Am Soc Echocardiogr. 2020;33(1):101‐109.e1. 31585830 10.1016/j.echo.2019.07.017

[14] Poh KK , Ton‐Nu TT , Neilan TG , Tournoux FB , Picard MH , Wood MJ . Myocardial adaptation and efficiency in response to intensive physical training in elite speedskaters. Int J Cardiol. 2008;126(3):346‐351. 17602763 10.1016/j.ijcard.2007.04.051

[15] Nistri S , Galderisi M , Ballo P , et al. Determinants of echocardiographic left atrial volume: implications for normalcy. Eur J Echocardiogr. 2011;12(11):826‐833. 21880608 10.1093/ejechocard/jer137

[16] D'Andrea A , Riegler L , Cocchia R , et al. Left atrial volume index in highly trained athletes. Am Heart J. 2010;159(6):1155‐1161. 20569734 10.1016/j.ahj.2010.03.036

[17] Legaz Arrese A , Serrano Ostáriz E , González Carretero M , Lacambra Blasco I . Echocardiography to measure fitness of elite runners. J Am Soc Echocardiogr. 2005;18(5):419‐426. 15891751 10.1016/j.echo.2005.02.002

[18] Hoogsteen J , Hoogeveen A , Schaffers H , et al. Left atrial and ventricular dimensions in highly trained cyclists. Int J Cardiovasc Imaging. 2003;19(3):211‐217. 12834157 10.1023/a:1023684430671

[19] Csanády M , Forster T , Högye M . Comparative echocardiographic study of junior and senior basketball players. Int J Sports Med. 1986;7(3):128‐132. 2942498 10.1055/s-2008-1025749

[20] Cavarretta E , Maffessanti F , Sperandii F , et al. Reference values of left heart echocardiographic dimensions and mass in male peri‐pubertal athletes. Eur J Prev Cardiol. 2018;25(11):1204‐1215. 29775081 10.1177/2047487318776084

[21] Somauroo JD , Pyatt JR , Jackson M , Perry RA , Ramsdale DR . An echocardiographic assessment of cardiac morphology and common ECG findings in teenage professional soccer players: reference ranges for use in screening. Heart. 2001 Jun;85(6):649‐654. 11359746 10.1136/heart.85.6.649 PMC1729780

[22] Gjerdalen GF , Hisdal J , Solberg EE , Andersen TE , Radunovic Z , Steine K . Atrial size and function in athletes. Int J Sports Med. 2015;36(14):1170‐1176. 26509381 10.1055/s-0035-1555780

[23] Wilhelm M , Brem MH , Rost C , et al. Early repolarization, left ventricular diastolic function, and left atrial size in professional soccer players. Am J Cardiol. 2010;106(4):569‐574. 20691318 10.1016/j.amjcard.2010.03.072

[24] Nishimura T , Yamada Y , Kawai C . Echocardiographic evaluation of long‐term effects of exercise on left ventricular hypertrophy and function in professional bicyclists. Circulation. 1980;61(4):832‐840. 6444559 10.1161/01.cir.61.4.832

[25] Elliott AD , Mahajan R , Linz D , et al. Atrial remodeling and ectopic burden in recreational athletes: implications for risk of atrial fibrillation. Clin Cardiol. 2018;41(6):843‐848. 29671875 10.1002/clc.22967 PMC6490079

[26] Pelliccia A , Kinoshita N , Pisicchio C , et al. Long‐term clinical consequences of intense, uninterrupted endurance training in olympic athletes. J Am Coll Cardiol. 2010;55(15):1619‐1625. 20378081 10.1016/j.jacc.2009.10.068

[27] Aurigemma GP , Gottdiener JS , Arnold AM , Chinali M , Hill JC , Kitzman D . Left atrial volume and geometry in healthy aging: the cardiovascular health study. Circ Cardiovasc Imaging. 2009;2(4):282‐289. 19808608 10.1161/CIRCIMAGING.108.826602 PMC4156514

[28] Thomas L , Levett K , Boyd A , Leung DYC , Schiller NB , Ross DL . Compensatory changes in atrial volumes with normal aging: is atrial enlargement inevitable? J Am Coll Cardiol. 2002;40(9):1630‐1635. 12427416 10.1016/s0735-1097(02)02371-9

[29] Gjerdalen GF , Hisdal J , Solberg EE , Andersen TE , Radunovic Z , Steine K . The Scandinavian athlete's heart; echocardiographic characteristics of male professional football players. Scand J Med Sci Sports. 2014;24(5):e372‐e380. 24472028 10.1111/sms.12178

[30] Riding NR , Salah O , Sharma S , et al. ECG and morphologic adaptations in Arabic athletes: are the European Society of Cardiology's recommendations for the interpretation of the 12‐lead ECG appropriate for this ethnicity? Br J Sports Med. 2014;48(15):1138‐1143. 23564906 10.1136/bjsports-2012-091871

[31] Crouse SF , White S , Erwin JP , et al. Echocardiographic and blood pressure characteristics of first‐year collegiate American‐style football players. Am J Cardiol. 2016;117(1):131‐134. 26554673 10.1016/j.amjcard.2015.09.049

[32] D'Ascenzi F , Pelliccia A , Natali BM , et al. Morphological and functional adaptation of left and right atria induced by training in highly trained female athletes. Circ Cardiovasc Imaging. 2014;7(2):222‐229. 24470314 10.1161/CIRCIMAGING.113.001345

[33] Jegier W , Sekelj P , Auld PA , et al. The relation between cardiac output and body size. Br Heart J. 1963;25:425‐430. 14045321 10.1136/hrt.25.4.425 PMC1018015

[34] Batterham AM , George KP , Whyte G , Sharma S , McKenna W . Scaling cardiac structural data by body dimensions: a review of theory, practice, and problems. Int J Sports Med. 1999;20(8):495‐502. 10606211 10.1055/s-1999-8844

[35] Gutgesell HP , Rembold CM . Growth of the human heart relative to body surface area. Am J Cardiol. 1990;65(9):662‐668. 2309636 10.1016/0002-9149(90)91048-b

[36] D'Ascenzi F , Solari M , Anselmi F , et al. Atrial chamber remodelling in healthy pre‐adolescent athletes engaged in endurance sports: a study with a longitudinal design. The CHILD study. Int J Cardiol. 2016;223:325‐330. 27543703 10.1016/j.ijcard.2016.08.231

[37] McClean G , George K , Lord R , et al. Chronic adaptation of atrial structure and function in elite male athletes. Eur Heart J Cardiovasc Imaging. 2015;16(4):417‐422. 25368211 10.1093/ehjci/jeu215

[38] Moro AS , Okoshi MP , Padovani CR , Okoshi K . Doppler echocardiography in athletes from different sports. Med Sci Monit. 2013;19:187‐193. 23478754 10.12659/MSM.883829 PMC3628709

[39] Claessen G , Colyn E , La Gerche A , et al. Long‐term endurance sport is a risk factor for development of lone atrial flutter. Heart. 2011;97(11):918‐922. 21398696 10.1136/hrt.2010.216150

[40] Barbier J , Lebiller E , Ville N , Rannou‐Bekono F , Carré F . Relationships between sports‐specific characteristics of athlete's heart and maximal oxygen uptake. Eur J Cardiovasc Prev Rehabil. 2006;13(1):115‐121. 16449874 10.1097/00149831-200602000-00018

[41] Ikäheimo MJ , Palatsi IJ , Takkunen JT . Noninvasive evaluation of the athletic heart: sprinters versus endurance runners. Am J Cardiol. 1979;44(1):24‐30. 156493 10.1016/0002-9149(79)90246-7

[42] Kooreman Z , Giraldeau G , Finocchiaro G , et al. Athletic remodeling in female college athletes: the "Morganroth hypothesis" revisited. Clin J Sport Med. 2019;29(3):224‐231. 31033616 10.1097/JSM.0000000000000501

[43] Date H , Imamura T , Onitsuka H , et al. Differential increase in natriuretic peptides in elite dynamic and static athletes. Circ J. 2003;67(8):691‐696. 12890912 10.1253/circj.67.691

[44] Toufan M , Kazemi B , Akbarzadeh F , Ataei A , Khalili M . Assessment of electrocardiography, echocardiography, and heart rate variability in dynamic and static type athletes. Int J Gen Med. 2012;5:655‐660. 22924010 10.2147/IJGM.S33247 PMC3422899

[45] D'Andrea A , Riegler L , Golia E , et al. Range of right heart measurements in top‐level athletes: the training impact. Int J Cardiol. 2013;164(1):48‐57. 21737163 10.1016/j.ijcard.2011.06.058

[46] D'Andrea A , Cocchia R , Riegler L , et al. Aortic root dimensions in elite athletes. Am J Cardiol. 2010;105(11):1629‐1634. 20494674 10.1016/j.amjcard.2010.01.028

[47] Cuspidi C , Sala C , Tadic M , et al. Left atrial volume in elite athletes: a meta‐analysis of echocardiographic studies. Scand J Med Sci Sports. 2019;29(7):922‐932. 30866082 10.1111/sms.13416

[48] Mansencal N , Marcadet DM , Martin F , Montalvan B , Dubourg O . Echocardiographic characteristics of professional tennis players at the Roland Garros French Open. Am Heart J. 2007;154(3):527‐531. 17719301 10.1016/j.ahj.2007.04.056

[49] Smekal G , von Duvillard SP , Rihacek C , et al. A physiological profile of tennis match play. Med Sci Sports Exerc. 2001;33(6):999‐1005. 11404666 10.1097/00005768-200106000-00020

[50] Wilhelm M , Roten L , Tanner H , et al. Long‐term cardiac remodeling and arrhythmias in nonelite marathon runners. Am J Cardiol. 2012;110(1):129‐135. 22459307 10.1016/j.amjcard.2012.02.058

[51] Grimsmo J , Grundvold I , Maehlum S , Arnesen H . Echocardiographic evaluation of aged male cross country skiers. Scand J Med Sci Sports. 2011;21(3):412‐419. 20136762 10.1111/j.1600-0838.2009.01054.x

[52] Pelliccia A , Maron BJ , Di Paolo FM , et al. Prevalence and clinical significance of left atrial remodeling in competitive athletes. J Am Coll Cardiol. 2005;46(4):690‐696. 16098437 10.1016/j.jacc.2005.04.052

[53] Uberoi A , Sadik J , Lipinski MJ , van le V , Froelicher V . Association between cardiac dimensions and athlete lineup position: analysis using echocardiography in NCAA football team players. Phys Sportsmed. 2013;41(3):58‐66. 10.3810/psm.2013.09.2025 24113703

[54] Dores H , Mendes L , Dinis P , Cardim N , Monge JC , Santos JF . Myocardial deformation and volume of exercise: a new overlap between pathology and athlete's heart? Int J Cardiovasc Imaging. 2018;34(12):1869‐1875. 30008149 10.1007/s10554-018-1412-3

[55] Gabrielli L , Herrera S , Contreras‐Briceño F , et al. Increased active phase atrial contraction is related to marathon runner performance. Eur J Appl Physiol. 2018;118(9):1931‐1939. 29971492 10.1007/s00421-018-3927-7

[56] Kayali S , Yildirim FT . Echocardiographic assessment of children participating in regular sports training. North Clin Istanb. 2018;6(3):236‐241. 31650109 10.14744/nci.2018.40360 PMC6790927

[57] Mahjoub H , Le Blanc O , Paquette M , et al. Cardiac remodeling after six weeks of high‐intensity interval training to exhaustion in endurance‐trained men. Am J Physiol Heart Circ Physiol. 2019;317(4):H685‐H694. 31347913 10.1152/ajpheart.00196.2019

[58] Król W , Jędrzejewska I , Konopka M , et al. Left atrial enlargement in young high‐level endurance athletes ‐ another sign of athlete's heart? J Hum Kinet. 2016;53:81‐90. 28149413 10.1515/hukin-2016-0012 PMC5260578

[59] Rundqvist L , Engvall J , Faresjö M , Carlsson E , Blomstrand P . Regular endurance training in adolescents impacts atrial and ventricular size and function. Eur Heart J Cardiovasc Imaging. 2017;18(6):681‐687. 27406576 10.1093/ehjci/jew150

[60] Saito K , Matushita M . The contribution of left ventricular mass to maximal oxygen uptake in female college rowers. Int J Sports Med. 2004;25(1):27‐31. 14750009 10.1055/s-2003-45229

[61] Hedman K , Tamás É , Henriksson J , Bjarnegård N , Brudin L , Nylander E . Female athlete's heart: systolic and diastolic function related to circulatory dimensions. Scand J Med Sci Sports. 2015;25(3):372‐381. 24840312 10.1111/sms.12246

[62] D'Ascenzi F , Cameli M , Lisi M , et al. Left atrial remodelling in competitive adolescent soccer players. Int J Sports Med. 2012;33(10):795‐801. 22562745 10.1055/s-0032-1304660

[63] Saito K , Matsushita M , Matsuo A . The effects of training on left ventricular systolic and diastolic function in female college rowers. Jpn Heart J. 1998;39(4):411‐417. 9810292 10.1536/ihj.39.411

[64] Shah AB , Zilinski J , Brown MG , et al. Endurance exercise training attenuates natriuretic peptide release during maximal effort exercise: biochemical correlates of the "athlete's heart". J Appl Physiol. 2018;1702‐1709. [Epub ahead of print] 10.1152/japplphysiol.00293.2018. 30307785

[65] D'Ascenzi F , Pelliccia A , Natali BM , et al. Training‐induced dynamic changes in left atrial reservoir, conduit, and active volumes in professional soccer players. Eur J Appl Physiol. 2015;115(8):1715‐1723. 25808863 10.1007/s00421-015-3151-7

[66] Pedlar CR , Brown MG , Shave RE , et al. Cardiovascular response to prescribed detraining among recreational athletes. J Appl Physiol. 2018;124(4):813‐820. 29212672 10.1152/japplphysiol.00911.2017

[67] Brugger N , Krause R , Carlen F , et al. Effect of lifetime endurance training on left atrial mechanical function and on the risk of atrial fibrillation. Int J Cardiol. 2014;170(3):419‐425. 24342396 10.1016/j.ijcard.2013.11.032

[68] Sanchis‐Gomar F , Garatachea N , Catalán P , López‐Ramón M , Lucia A , Serrano‐Ostáriz E . LA Size in Former Elite Athletes. JACC Cardiovasc Imaging. 2016;9(5):630‐632. 10.1016/j.jcmg.2015.08.023 26897686

[69] Luthi P , Zuber M , Ritter M , et al. Echocardiographic findings in former professional cyclists after long‐term deconditioning of more than 30 years. Eur J Echocardiogr. 2008;9(2):261‐267. 17470417 10.1016/j.euje.2007.03.001

[70] Höglund C . Enlarged left atrial dimension in former endurance athletes: an echocardiographic study. Int J Sports Med. 1986;7(3):133‐136. 2942499 10.1055/s-2008-1025750

[71] Hagmar M , Hirschberg AL , Lindholm C , et al. Athlete's heart in postmenopausal former elite endurance female athletes. Clin J Sport Med. 2005;15(4):257‐262. 16003041 10.1097/01.jsm.0000171257.54908.e7

[72] Hasdemir H , Yildiz M , Metin G , et al. Aortic properties and atrial electrophysiology in the young and old football players. Rev Assoc Med Bras. 2011;57(3):280‐285. 21691690 10.1590/s0104-42302011000300009

[73] Pelliccia A , Maron BJ , de Luca R , et al. Remodeling of left ventricular hypertrophy in elite athletes after long‐term deconditioning. Circulation. 2002;105(8):944‐949. 11864923 10.1161/hc0802.104534

[74] Rodevan O , Bjornerheim R , Ljosland M , et al. Left atrial volumes assessed by three‐ and two‐dimensional echocardiography compared to MRI estimates. Int J Card Imaging. 1999;15(5):397‐410. 10595406 10.1023/a:1006276513186

[75] Gardner BI , Bingham SE , Allen MR , Blatter DD , Anderson JL . Cardiac magnetic resonance versus transthoracic echocardiography for the assessment of cardiac volumes and regional function after myocardial infarction: an intrasubject comparison using simultaneous intrasubject recordings. Cardiovasc Ultrasound. 2009;7:38. 19689809 10.1186/1476-7120-7-38 PMC2743646

[76] Badano LP , Kolias TJ , Muraru D , et al. Standardization of left atrial, right ventricular, and right atrial deformation imaging using two‐dimensional speckle tracking echocardiography: a consensus document of the EACVI/ASE/Industry Task Force to standardize deformation imaging. Eur Heart J Cardiovasc Imaging. 2018;19(6):591‐600. 29596561 10.1093/ehjci/jey042

[77] Cuspidi C , Tadic M , Sala C , Gherbesi E , Grassi G , Mancia G . Left atrial function in elite athletes: a meta‐analysis of two‐dimensional speckle tracking echocardiographic studies. Clin Cardiol. 2019;42(5):579‐587. 30907013 10.1002/clc.23180 PMC6523010

[78] Kawakami H , Ramkumar S , Pathan F , et al. Use of echocardiography to stratify the risk of atrial fibrillation: comparison of left atrial and ventricular strain. Eur Heart J Cardiovasc Imaging. 2020;21(4):399–407. 31578558 10.1093/ehjci/jez240

[79] Saha SK , Anderson PL , Caracciolo G , et al. Global left atrial strain correlates with CHADS2 risk score in patients with atrial fibrillation. J Am Soc Echocardiogr. 2011;24(5):506‐512. 21477990 10.1016/j.echo.2011.02.012

[80] Hubert A , Galand V , Donal E , et al. Atrial function is altered in lone paroxysmal atrial fibrillation in male endurance veteran athletes. Eur Heart J Cardiovasc Imaging. 2018;19(2):145‐153. 29069358 10.1093/ehjci/jex225

